# Multifaceted Role of the Placental Growth Factor (PlGF) in the Antitumor Immune Response and Cancer Progression

**DOI:** 10.3390/ijms20122970

**Published:** 2019-06-18

**Authors:** Loredana Albonici, Maria Gabriella Giganti, Andrea Modesti, Vittorio Manzari, Roberto Bei

**Affiliations:** Department of Clinical Sciences and Translational Medicine, University of Rome “Tor Vergata”, Via Montpellier 1, 00133 Rome, Italy; giganti@med.uniroma2.it (M.G.G.); modesti@med.uniroma2.it (A.M.); manzari@med.uniroma2.it (V.M.); bei@med.uniroma2.it (R.B.)

**Keywords:** PlGF, VEGF, VEGFR, cancer progression

## Abstract

The sharing of molecules function that affects both tumor growth and neoangiogenesis with cells of the immune system creates a mutual interplay that impairs the host’s immune response against tumor progression. Increasing evidence shows that tumors are able to create an immunosuppressive microenvironment by recruiting specific immune cells. Moreover, molecules produced by tumor and inflammatory cells in the tumor microenvironment create an immunosuppressive milieu able to inhibit the development of an efficient immune response against cancer cells and thus fostering tumor growth and progression. In addition, the immunoediting could select cancer cells that are less immunogenic or more resistant to lysis. In this review, we summarize recent findings regarding the immunomodulatory effects and cancer progression of the angiogenic growth factor namely placental growth factor (PlGF) and address the biological complex effects of this cytokine. Different pathways of the innate and adaptive immune response in which, directly or indirectly, PlGF is involved in promoting tumor immune escape and metastasis will be described. PlGF is important for building up vascular structures and functions. Although PlGF effects on vascular and tumor growth have been widely summarized, its functions in modulating the immune intra-tumoral microenvironment have been less highlighted. In agreement with PlGF functions, different antitumor strategies can be envisioned.

## 1. Introduction

To date, important strategies against tumor growth include antiangiogenic therapies and activation or manipulation of the host’s immune system against tumor cells [1,2]. Antiangiogenic therapy, such as the use of vascular endothelial growth factor (VEGF) pathways inhibitors, has been shown to elicit only a transient tumor and vascular shrinkage followed by enhanced tumor invasion and metastases [3,4,5,6,7,8,9]. In addition, rising evidence has supported the suppressive role of VEGF signaling in T cells functions, such as the reduction of the cytotoxic activity of CD8 cells [10,11,12,13,14].

On the other hand, natural or induced immunotherapeutic approaches (e.g., treatment with vaccines, antibodies blocking inhibitory receptors, and antibodies agonizing costimulatory receptors) or synthetically use host’s immune system for an effective antitumor immune response, are not enough potent or always effective. Indeed, in most cancers, immune cells infiltrating tumors often show an aberrant phenotype that reflects the weakness of the antitumor activity and favors tumor growth. Both the tumor-induced polarization of immune cells and the selection of cancer cells resistant to immune attack, contribute to the tumor immune escape [15,16,17,18,19,20,21,22].

Several pieces of evidence have revealed that the immune-mediated mechanisms are able to regulate the response to antiangiogenic therapy and that drugs having anti-angiogenic properties can counteract the tumor-induced immunosuppression [23,24,25,26,27,28].

Therefore, it appears reasonable to assume that the progression of the tumor could be effectively reduced by combining antiangiogenic strategies with therapies that reduce pro-tumor immune-based effectors or neutralize those that counteract the development of anti-tumor immunity, thus providing synergistic effects between antiangiogenic therapy and immune-modulatory approaches.

## 2. Immunosurveillance and Immunoediting in Cancer

Early last century, Paul Ehrlich proposed that the incidence of cancer would be much greater if the immune system failed in identifying and eliminating nascent tumor cells [29]. Fifty years later Thomas and Burnet suggested that immune T cells were the pivotal sentinel in the immune system’s response against cancer [30,31]. This idea led to the coinage of the term “immunosurveillance” which describes the concept whereby the immune system is on perpetual alert against transformed cells. This concept formally envisaged that adaptive immunity was responsible for preventing cancer development in immunocompetent hosts. Nonetheless, this concept was later questioned since no differences were found between athymic nude mice and syngeneic wild-type mice in tumor development [32,33].

However, the role of immunosurveillance in cancer has been fully elucidated by experimental and clinical studies, only at the beginning of this century. These studies have revealed that the immune system can play a critical role in maintaining an equilibrium between immune recognition and tumor development by promoting or suppressing tumor growth. In this regard, Shankaran et al. have experimentally unveiled the process of immunoediting through the demonstration of the increased incidence of malignant tumors in immune-deficient mice. They reported that mice deficient for lymphocytes (RAG 2-deficient mice) or interferon (IFN) type I receptor signaling were more susceptible to carcinogen-induced sarcomas and spontaneous epithelial carcinomas development than wild-type mice. However, the ability of malignant tumors to develop in patients with a fully functioning immune system suggested the term “cancer immunoediting” to describe the constant protective and sculpting actions of the immune response on developing tumors. [34]. This concept is based on the idea that the immune system, not only protects the host by tumor development, but it shapes the immunogenicity of the tumor as well [35,36,37,38]. This hypothesis is now considered a new “hallmark of cancer” [39]. Thus, either the immune system can eliminate tumor cells expressing specific antigens or, in cases where the immune system is not able to eradicate cancer, a state of equilibrium develops whereby the tumor does not progress or further metastasize. Alternatively, if the immune response fails to eliminate the tumor, cancer cells that can resist, elude, or suppress the antitumor immune response are selected, leading to the tumor escape and a progressively growing tumor.

Later, transplantation experiments showed that cancer cells that originally arose in immunodeficient mice are often inefficient at initiating secondary tumors in syngeneic immunocompetent hosts, whereas cancer cells from tumors arising in immunocompetent mice are equally efficient at initiating transplanted tumors in both types of hosts [40]. Thus, the immune response can function as an effective tumor-suppressor system, as confirmed by the evidence that tumor infiltration by CD8^+^ cytotoxic T lymphocytes (CTL) or natural killer (NK) cells is correlated with a better prognosis in patients with melanoma, liver, breast or colon cancer [41,42,43,44,45].

Accumulating evidence has recognized the importance of the host immune system in controlling tumor progression and ensuring the clinical outcome among tumor-bearing patients within the same stage of TNM-classification.

Detailed intra-tumor analysis has revealed that immune infiltrates are very heterogeneous, may reflect distinct underlying biology of the tumor and can significantly vary in tumors within the same stage and in different patients with the same type of tumor. Thus, the location, abundance and function of different immune cell populations [including macrophages, dendritic cells (DC), NK cells, B cells and *naïve* and memory T lymphocytes (which include Th1, Th2, Th17, Treg, and CTL)], termed as the “immune contexture” [46], have allowed the identification of components that are either beneficial or deleterious for cancer patients. The immune contexture includes the innate immune activation, the recruitment of immune cells by chemokines, the activation of immune effector molecules and the expression of immunoregulatory factors [46]. Therefore, the immunophenotyping of tumors has been proposed as new tool termed “Immunoscore” that might be included into traditional TNM classification, designated as TNM-I (TNM-Immune), thus providing an essential prognostic and potentially predictive tool [47,48]. Finally, the understanding of the complexity and heterogeneity of the immune context of the tumor microenvironment has been considered, able to affect the response to therapy [49].

Thus, paradoxically, the immune response can not only suppress tumor growth but can also promote tumor progression either by selecting tumor cells that are more suitable to survive in an immunocompetent host or by establishing conditions within the tumor microenvironment that facilitate tumor progression [50,51,52,53,54]. Indeed, mounting evidence has shown that the tumor microenvironment can prevent the activation and the expansion of specific anti-tumor helper and cytotoxic T cells and simultaneously promote the production of pro-inflammatory cytokines and other molecules, leading to the accumulation of immune suppressive cells that inhibit rather than promote immunity. Among the other, tumor-associated macrophages (TAMs), myeloid-derived suppressor cells (MDSC) and regulatory T (Treg) lymphocytes are present within the tumor microenvironment [50,51,52,53,54,55,56,57,58]. In fact, malignant progression is accompanied by an extensive immune suppression that interferes with an effective antitumor response [16,59,60,61,62]. Although changes in tumor cells (loss of MHC molecules, loss of tumor antigens, loss of complement or T cellular lysis or NK cell sensitivity) make them a defective target for the immune attack, it is now widely accepted that immunosuppression is primarily the result of the ability of tumor cells to subvert the immune system to their advantage [50,58,60,61,62]. Infiltration of tumors by the recruitment of inflammatory/myeloid-derived cells can result in a state of unresolved immune responses, such as chronic inflammation, that maintains and promotes cancer progression and suppresses the anticancer immune response [63,64,65,66,67].

## 3. The Interplay between Angiogenic Growth Factors and Immune Cells

The link between cancer and inflammation as well as cancer and angiogenesis has been widely reported. However, while many authors have focused on the impact of the immune system in modulating angiogenesis in the tumor microenvironment, thus allowing tumor growth and invasiveness, less attention has been paid to the effects that angiogenic growth factors may play on regulating the immune response during the tumor promotion and progression.

Angiogenesis is a process that is involved in the formation of new blood vessels from preexisting ones and represents a key event in the development of tumors since the vascular system provides the supply of oxygen and nutrients to cancer cells and the elimination of waste. In the absence of oxygen, hypoxia induces the expression of transcriptional factors such as hypoxia-inducible factor (HIF) in the center of the tumor. HIF, in turn, upregulates several pro-angiogenic factors including placental growth factor (PlGF) and vascular endothelial growth factor (VEGF) [68,69,70,71]. High levels of VEGF during tumor growth have been associated with an immunodeficiency state.

It is interesting to note that many if not all angiogenic factors also directly or indirectly mediate the inflammatory response and are able to activate immune cells. Among the angiogenic growth factors upregulated during inflammation and tumor development and progression, a controversial role has been attributed to PlGF.

## 4. Biological Effects of Placental Growth Factor (PlGF)

PlGF is a 25-kd pleiotropic cytokine, originally discovered in human placenta [72], which belongs to the vascular endothelial growth factor (VEGF) family that also includes VEGF-A (from here also referred to as VEGF), VEGF-B, VEGF-C, VEGF-D and VEGF-E.

PlGF is a homodimeric glycoprotein encoded by a single PlGF gene that gives rise to four splice isoforms in humans, PlGF-1 (PlGF131), PlGF-2 (PlGF152), PlGF-3 (PlGF203) and PlGF-4 (PlGF224). PlGF-1 and PlGF-2 represent the predominant isoforms [73,74,75]. Only one form, equivalent to human PlGF-2, is expressed in mice [76]. PlGF-1 and PlGF-3 are diffusible isoforms, while PlGF-2 and PlGF-4 have heparin-binding domains.

Unlike VEGF, PlGF homodimers bind VEGFR1, but not VEGFR2 [77]. However, only PlGF-2 and PlGF-4 are able to bind the co-receptors neuropilin-1 (NRP1), neuropilin-2 (NRP2) and heparan sulfate proteoglycans (HSPG) due to the insertion of 21 basic amino acids [78,79,80]. Several stimuli ranging from hypoxia, inflammatory cytokines, growth factors, hormones, and oncogenes, are able to upregulate the expression of PlGF in disease conditions. Hypoxia upregulates the PlGF receptor VEGFR1 and its coreceptor NRP1 in disease conditions as well [77,81].

However, PlGF has been studied less than VEGF because it is not essential for the normal murine development and because PlGF binds only to VEGFR1 and not to VEGFR2, the latter being considered the main signaling receptor in angiogenesis. Unlike VEGF, PlGF plays a negligible role in the development and physiological angiogenesis and is not required as a survival signal for the maintenance of quiescent vessels in healthy tissues. Indeed, the genetic ablation of PlGF does not alter the vascular morphogenesis, adult *plgf -/-* mice are apparently healthy, fertile and without vascular defects [82,83,84].

**Figure 1:**
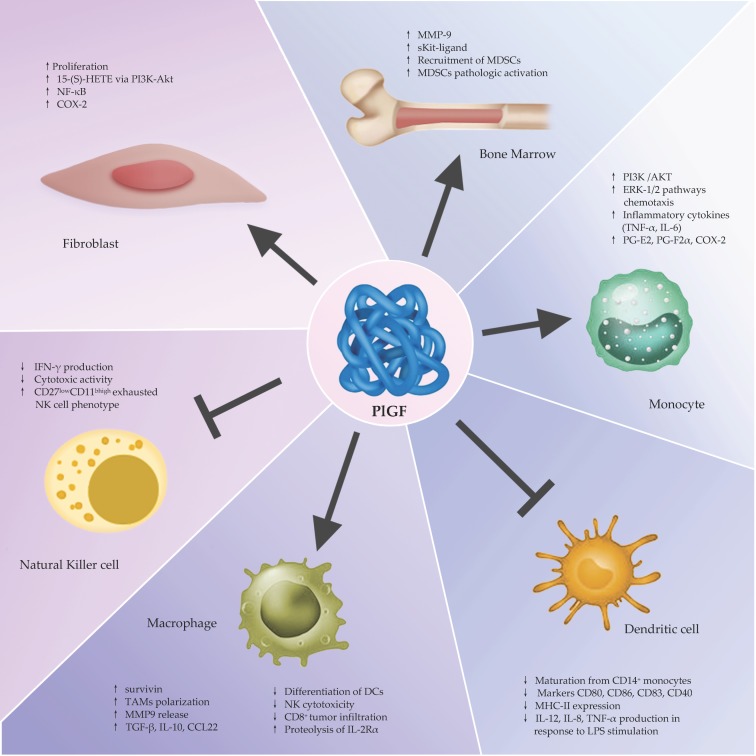
Pleotropic effects of placental growth factor. PlGF promotes the recruitment of VEGFR1^+^ hematopoietic stem cells (HSC), stimulates the recruitment and/or activation (e.g., cytokines production) of monocytes and activates macrophages, enhances the proliferation and survival of macrophages, blunts the antitumor immune response and stimulates the proliferation of mesenchymal fibroblasts and recruits myeloid progenitors to growing sprouts and collateral vessels. Abbreviations: PI3K, phosphoinositide 3-kinase; ERK, extracellular signal–regulated kinase, PG-E2, prostaglandin E2; PG-F2α, prostaglandin F2α; COX-2, cicloxigenase-2; MHC-II, major histocompatibility complex-II; LPS, lipopolysaccharides; DCs, dendritic cells; IL-2 Rα, IL-2 high affinity receptor; MMP9, matrix metallopeptidase 9; TAMs, tumor associated macrophages; 15-(S)-HETE, 15-Hydroxyeicosatetraenoic acid.

PlGF exerts different non-redundant effects in stress conditions including wound healing, tissue ischemia and sepsis [85]. Indeed, PlGF can have both direct effects on endothelial cells and indirect effects on nonvascular cells with pro-angiogenic activity by modulating the behavior of immune cells. PlGF enhances the proliferation, migration, and survival of macrophages [86,87], attracts monocytes and activates macrophages capable of releasing angiogenic and lymphangiogenic factors [88,89,90,91,92]. PlGF has also a direct effect on the inflammatory reaction because, by binding to VEGFR1 on monocytes, triggers TNF-α and IL-6 production via a calcineurin-dependent pathway [89]. In addition, PlGF stimulates the proliferation of mesenchymal fibroblasts and recruits myeloid progenitors to grow sprouts and collateral vessels [82,93,94,95,96] (Figure 1).

Coherently with its pleiotropic action, PlGF stimulates keratinocyte migration in wound healing [97] and exerts a protective paracrine effect in the ischemic heart [98,99,100,101]. In addition, PlGF promotes survival of cortical neurons [102], stimulates the axon growth cone formation of dorsal root ganglion neurons [103] and supports proliferation and migration of Schwann cells [104] as well. Lastly, PlGF regulates the maturation of uterine NK cells in the endometrium needed for trophoblast invasion [105].

After all, PlGF is able to upregulate the expression of additional angiogenic factors such as VEGF, basic fibroblast growth factor (FGF-2), platelet-derived growth factor β (PDGF-β), and matrix metalloproteinases (MMPs), among other molecules, by periendothelial fibroblasts, smooth muscle cells, or inflammatory cells in wound or tumor stroma as well [106,107].

## 5. PlGF Receptors and Signaling

### 5.1. VEGFR1

PlGF transmits its own signal via VEGFR1, which is different from VEGF because it stimulates phosphorylation of specific VEGFR1 tyrosine residues leading to expression of distinct target genes [81,108,109]. VEGFR1 is expressed by endothelial cells (EC) as well as tumor cells, bone marrow-derived pro-angiogenic and pro-inflammatory cells (mostly monocyte-macrophage lineage cells), vascular smooth muscle cells and by stromal cells such as fibroblasts [110]. Since the tyrosine kinase activity of VEGFR1 is relatively weak, VEGFR1-specific ligands, such as PlGF and VEGF do not stimulate significantly the proliferation of vascular smooth muscle cells, primary endothelial cells, pericytes or fibroblasts in culture. For the reasons, it has been considered a decoy receptor in physiological conditions [109,110,111].

Nonetheless, PlGF may sustain VEGF activity through different mechanisms involving both VEGF receptors. Indeed, an increase in PlGF may displace VEGF from VEGFR1 and thereby increase the fraction of VEGF available to activate its main receptor VEGFR2. In cells that co-express both growth factors, naturally occurring PlGF/VEGF heterodimers have been isolated and shown to bind to VEGFR1/VEGFR2 receptor complexes, thus inducing receptor cross talk and activation of VEGFR2, the major mediator of VEGF activities. Alternatively, the activation of VEGFR1 by PlGF homodimers may indirectly induce activation of VEGFR2 through the intermolecular transphosphorylation of VEGFR2 by VEGFR1 that amplifies VEGF/VEGFR2 signaling [86]. Finally, PlGF is able to induce its own angiogenic signal through VEGFR1 independently of VEGF via the phosphatidylinositol 3-kinase (PI3K) pathway and the upregulation of Bcl-2 in endothelial cells [112,113,114,115].

Since VEGFR1 has been also implicated in the mobilization of bone marrow-derived cells [89,96,100], monocyte migration and chemotaxis [100,116], it has been proposed as a novel cell surface marker for the monocyte-macrophage lineage in humans [117]. PlGF binding to VEGFR-1 in monocytes, results in the activation of PI3 kinase/AKT and ERK-1/2 pathways, leading to chemotaxis and the induction of several inflammatory cytokines. Further, PlGF enhances the proliferation and survival of macrophages by upregulating the antiapoptotic gene survivin [89,118]. In addition to the phosphorylation of Akt and p38, VEGFR1 can increase STAT3 in response to PlGF in hypoxic cells as well [119] (Figure 1).

Although the downstream signaling of VEGFR1 is not fully understood, VEGFR1 is regarded as a negative regulator for angiogenesis during embryogenesis and physiological conditions. Indeed, *vegfr1-null* mutant mice show severe defects of the hematopoietic system and die early in embryogenesis due to the uncontrolled growth of endothelial cells and disorganization of the vascular architecture. These findings indicate that VEGFR1 plays a negative role by suppressing pro-angiogenic signals to establish a balance essential for physiological angiogenesis [120]. On the other hand, mice lacking the VEGFR1 tyrosine kinase domain (*Flt-1 TK−/−*) have a normal development but display impairment of macrophage function [120].

Since VEGFR1 can affect the development of the hematopoietic system, the loss of post-natal expression of VEGFR1 could be associated with the negative regulation of hematopoietic cell motility and ensuing monocytes recruitment. A further feature, in clarifying the role played by PlGF on the hematopoietic system, derives from the study of Hattori et al., which found that PlGF promotes the recruitment of VEGFR1^+^ hematopoietic stem cells (HSC) because it can restore both early and late phases of hematopoiesis following bone marrow suppression by radiation. They found that PlGF enhances early phases of bone marrow recovery directly through the rapid chemotaxis of VEGFR1^+^ progenitor cells. The late phase of hematopoietic recovery is driven by PlGF-induced upregulation of matrix metalloproteinase-9 (MMP9), which in turn allows the release of stem cell factor (SCF)/soluble Kit-ligand by stromal cells. Thus, PlGF promotes the recruitment of VEGFR1^+^ HSCs from a quiescent to a proliferative bone marrow microenvironment, favoring differentiation, mobilization, and reconstitution of hematopoiesis [93,121] (Figure 1).

Remarkably, VEGFR1 is also expressed by T lymphocytes. While VEGFR1 and VEGFR2 mRNA is transcribed in both resting and activated T cells, VEGFR1 is the only receptor expressed on the cell surface of activated T cells [122]. Again, although the VEGFR1 signaling does not affect the proliferation of T lymphocytes, the engagement of VEGFR1 on activated T cells induces such cells both to migrate towards the tumor site and to increase locally IL-10 production, indicating that VEGFR1 is able to mediate a direct immunomodulatory effect on T cells [122].

### 5.2. Neuropilins

The pleiotropic coreceptors NRP1 and NRP2 were initially characterized as receptors for class III Semaphorin (Sema3) family members. These coreceptors are expressed by neuronal and non-neuronal cells, including endothelial cells and immune cells, involved in axon guidance as well as angiogenesis and immune response. *Nrp1-deficient* mice die early in embryogenesis and display a variety of abnormalities in the establishment of the cardiovascular system and in neuronal guidance. These abnormalities are associated with defective endothelial cell migration rather than proliferation [123]. On the other hand, mice expressing a *mutant nrp1 receptor*, with cytoplasmic domain deletion, result in vascular abnormalities distinct from those of *null-nrp1* and, although angiogenesis was unaffected, the vascular patterning was disrupted as evidenced by a frequent crossing of arteries and veins in the retina [124].

Gene inactivation of *nrp2* results in a less severe and distinct neuronal phenotype than the *nrp1* mutation and involves alterations of the venous and lymphatic system, because NRP2 expression is restricted to veins and lymphatic endothelium [125].

NRP family members are transmembrane proteins that possess two calcium-binding “CUB” domains (a1, a2), two coagulation factor V/VIII homology domains (b1, b2), a MAM (meprin, tyrosine phosphatase domain) region (c), a single transmembrane segment and a short intracellular domain. [126]. The a1 domain has been shown to interact with the sema domain of Sema3 ligands. Additionally, the b1 and b2 domains physically interact to basic residues involved in binding heparin, HSPG and basic amino acid residues of PlGF and other several growth factors such as HGF, TGF-β, bFGF, PDGF [79,127].

Although further studies are needed to reveal the real mechanisms concerning PlGF and Sema3 binding to NRP1, it is noteworthy to note that Sema3A is reported to be inducible in both DCs and T cells and to be involved in negative regulation of T cell activation [128]. Since the b1 domain contains a specific pocket essential for binding to the C-terminal domain of both PlGF and Sema3 ligands [127], it is possible to envisage a form of competition between the two ligands, especially when PlGF is upregulated, such as during inflammation or tumor growth.

Despite the steady increase in the understanding of NRP1 functions and its target cells and tissues, the mechanisms by which NRP1 mediates the functions of diverse ligands in different cell types remain elusive. NRPs have a short cytoplasmic tail and were initially regarded as non-signaling receptors. A variety of studies have now demonstrated that NRP1 possess a C-terminal SEA (Ser-Glu-Ala) motif implicated in associating with the PDZ [PSD-95 (postsynaptic density 95), Dlg (discs large) and ZO-1 (zonula occludens 1)] domain able to enhance responses to several growth factors under physiological and pathological conditions [129,130].

Apart from vessels and axons, NRPs are expressed primarily by dendritic cells (DCs) and regulatory T cells (Tregs) [131,132] and exert mainly inhibitory effects. NRP1 has been identified as a DCs marker corresponding to the blood dendritic cell antigen 4 (BDCA4) of human plasmacytoid DCs (pDCs), [133]. Thus, NRP1 mediates interactions between activated DCs and resting T cells that are essential for the initiation of the primary immune response [134].

Accordingly, given that NRP1 was also constitutively expressed on the surface of CD4^+^CD25^high^ Treg cells, independently of their activation status, it had been initially proposed as a surface marker to distinguish Treg cells from both *naïve* and recently activated CD4^+^ non-regulatory T cells. [135]. However, this finding was revised by a subsequent study by Milpied et al., in which they report that, in contrast to murine Treg, human Foxp3^+^ Tregs do not specifically express NRP1. Thus, NRP1 should not be used as a specific surface marker for these cells in human. Since NRP1 expression can be induced on peripheral blood T cells upon activation, NRP1 might represent at most a novel activation marker for human T cells [136].

The alternative splicing of both VEGFR1 and NRP1 pre-mRNA produces soluble receptor isoforms (sVEGFR1 and sNRP1 respectively) that can bind to and inhibit the action of both PlGF and VEGF [137,138,139,140]. Excessive sVEGFR1 generated by the human placenta and released into the circulation leads to hypertension and proteinuria in preeclampsia [137,138]. In the same manner, sNRP1, functioning as natural ligand trap, inhibits the interaction of PlGF-2 and VEGF with their specific receptors and with membrane NRP1 expressed by tumor or normal cells [139].

In addition, sVEGFR1 that neutralizes PlGF and VEGF induces a similar phenotype as employing an anti-VEGF antibody in healthy animals [140,141,142,143] and non-apoptotic death in cancer cells [144]. When PlGF expression is minimal, sVEGFR1 acts as a VEGF trap to prevent excessive VEGFR2 activation in physiological conditions. As PlGF is upregulated in cancer and stromal cells, it leads to displacement of VEGF from sVEGFR1 and consequently increases its bioavailability and binding to VEGFR2, leading to transmission of proangiogenic signals. Thus, VEGFR1 may function either directly, through stimulation of its tyrosine kinase domain or indirectly, through its soluble isoform by reducing the availability of its ligands for other receptors [81].

## 6. Multifaceted Role PlGF/VEGFR-1/NRP1/NRP2 Signaling in Anti-Tumor Immunity and Cancer Progression

### 6.1. The Impact on the Innate Immunity

PlGF binding to VEGFR-1 induces inflammation via the transcription factor nuclear factor-*κ*B (NF-*κ*B). PlGF expression is in turn controlled by NF-*κ*B. PlGF induces the degradation of I*κ*B-α, thereby increasing the NF-*κ*B p65 DNA-binding activity in hypoxic human monocytes. PlGF significantly increases IL-6 and IL-8 secretion, cyclooxygenase-2 (Cox-2) expression, and consequent prostaglandin (PG)-E2 and PG-F2α release, and MMP-9 gene expression and enzyme production. [106,145]. Accordingly, PlGF significantly enhanced the magnitude and duration of TNF-α mRNA and protein production by TLR-7/8-activated monocytes and increased the subsequent production of TNF-α-independent inflammatory cytokines. This PlGF/TLR effect involved multiple inflammatory cytokines/chemokines involving the majority of TLR agonists. PlGF also enhances the phosphorylation of I*κ*-B kinase (IKK) in monocytes stimulated with the TLR-7/8 agonist R848, and IKK inhibition completely suppresses the PlGF effect [146]. All these findings indicate the existence of a direct and not fortuitous connection between the cells of the immune system and the effect of PlGF (Figure 1).

An additional link between inflammation and PlGF has been provided with the demonstration that TNF-α is upregulated by tumor-derived PlGF in myelomonocytic cells via the transcription factor Nuclear Factor of Activated T cells-1 (NFAT-1), which in turn contributes to PlGF-induced myelomonocytic cell recruitment [91], thus establishing a vicious circle that feeds tumor-associated inflammation.

NFAT-1 has been initially identified in lymphocytes, and has been reported to be expressed in activated but not resting T cells [147] and later is shown to be expressed in several immune and non-immune cell types. Of note, the NFAT family proteins are key regulators of T-cell development and function and regulate not only T cell activation and differentiation but the function of other immune cells, including DCs and B cells as well. However, NFAT isoforms are also active in tumor and in stromal cells within the tumor microenvironment including fibroblasts, endothelial cells and infiltrating immune cells. All these cell types can release and respond via VEGFR1 to PlGF in inflammatory conditions. Noteworthy, NFAT family proteins, are involved in cell transformation, proliferation, invasive migration, tumor cell survival, tumor angiogenesis and tumor-induced anergy of CD4^+^ T cells by mediating the expression of several inflammatory cytokines, as well [148,149,150].

In this regard, the role of NFAT during the antitumor immune response has been elucidated by studies on a murine model of melanoma. Interestingly, although NFAT-deficient (*nfat1-/-*) mice are reported to be more susceptible of transformation than wild-type counterparts, the inoculation of *nfat1-/-* mice with B16F10 melanoma cells sustained less tumor growth in the lung than the wild-type counterparts. Even though melanoma cells equally colonized *nfat1-/-* and wild-type lungs, tumors did not progress in the absence of NFAT1 expression [150]. Remarkable, the expression of NFAT-1 in wild-type mice was associated to a massive mononuclear perivascular infiltrate of lungs, suggesting a critical role mediated by tumor-infiltrating immune and stromal cells and therefore suggestive of a soluble factor capable of recruiting mononuclear cells such PlGF. The mechanism by which NFAT could affect the immune response could be through the expression of anergy-associated genes, which results in defective production of several key effector cytokines in the context of cancer. Indeed, clonal anergy of CD4^+^ T cells occurs when the T cell receptor is activated in the absence of a co-stimulatory signal. Conversely, NFAT1 deficiency blunted the induction of anergy in tumor antigen-specific CD4^+^ T cells, thus enhancing anti-tumor responses [149,151]. Interestingly, Jinnin et al. reported that a region of the VEGFR1 promoter contains a binding site for the transcription factor NFAT, providing the first evidence that VEGFR1 represents a NFAT target gene [152]. Thus, PlGF could contribute to induce a state of immunosuppression, via NFAT, by binding the VEGFR1.

### 6.2. PlGF Promotes the Intratumor Immunosoppressive Microenvironment

Immunosuppression within the tumor microenvironment is the result of mechanisms exerted by tumor cells through their ability to recruit immunosuppressive cells into the tumor milieu. In fact, the cytokines and soluble factors found within the tumor microenvironment, create a diffuse tolerogenic environment that alters the normal hematopoiesis, favoring the expansion and recruitment of immature myeloid cells and, at the same time, preventing their local differentiation [18].

The main immune cell types contributing to tumor immunosuppression and escape are myeloid regulatory cells, which include tumor-associated macrophages (TAMs), type 2 natural killer T (NKT) cells, myeloid-derived suppressor cells (MDSCs) and regulatory T cells (Tregs) [153,154]. Features common to all MDSCs are the immature state and a remarkable ability to suppress T-cell responses. In addition to their suppressive effects on adaptive immune responses, MDSCs have also been reported to regulate innate immune responses through modulating cytokines production by macrophages [153,155]. Although the expression of overlapping cell surface markers has made it difficult to distinguish among different myeloid cell populations, MDSCs are reported as cells, that express the common myeloid marker CD33, but lack expression of markers of mature myeloid and lymphoid cells [153]. Further, this heterogeneous cell population is characterized by the increased production of reactive oxygen and nitrogen species, indoleamine 2,3-dioxygenase (IDO), arginase 1, TGF-β, IL10 and COX2 and depletion of cysteine, all potent suppressors of various T-cell functions, although these mechanisms are not simultaneously functioning [155,156,157].

MDSCs arise from a common myeloid precursor (CMP). Normal MDSCs activation is characterized by the production of mature myeloid cells (mostly macrophages) that are able to support the remodeling of tissues after resolved inflammation [157]. Conversely, MDSCs pathologic activation is the result of persistent stimulation of the myeloid compartment with relatively low-strength signals arising from tumor cells or sites of chronic inflammation [158]. Thus, myeloid cells produced under these conditions are unable to differentiate into mature cells, have poorly phagocytic activity and produce high levels of ROS, NO and mostly anti-inflammatory cytokines [159]. As a result, these cells acquire potent immune-suppressive potential and the tumor amplifies and takes advantage of this behavior to protect themselves from elimination by the immune system, but also in limiting the effect of immunotherapeutics [92,160,161].

#### 6.2.1. PlGF-Mediated Mechanisms of TAMs Polarization/Accumulation

Macrophages have an essential role in the innate defense mechanisms and can promote a specific immunity by inducing T cell recruitment and activation and by B cell interaction. Macrophages are characterized by functional plasticity, consequently, their activation can be either pro-inflammatory or anti-inflammatory and has been classified as “classical” and “alternative” or M1 and M2, respectively. Unlike M1 macrophages polarization, triggering Th1 adaptive immune reactions possesses inflammatory properties and promotes tissue cell destruction, the M2 macrophages, which suppress Th1 immunity, have anti-inflammatory properties and contribute to tissue remodeling, angiogenesis and wound healing [161].

Macrophages are also important components of the inflammatory microenvironment of tumors and represent a dominant myeloid population in many solid tumors [161,162,163]. The recruitment of macrophages in the tumor microenvironment is a receptor-dependent process and is regulated by chemotactic factors that are expressed by tumor endothelium, tumor, and stromal cells, and is regulated to a large degree by tumor hypoxia. Macrophages migrate to accumulate in the most severely hypoxic regions of tumor tissue [164,165,166]. Hypoxia strongly increases macrophage-mediated T-cell suppression in vitro, in a fashion dependent HIF-1α macrophage expression. Thus, the innate immune hypoxic response is linked to tumor progression through the induction of T-cell suppression in the tumor microenvironment [19]. In fact, the loss of HIF-1α expression in myeloid cells directly attenuates a hypoxia-induced suppression of T-cell activation and reduces tumor progression. Moreover, T cells derived from tumors in by myeloid *hif-1α–null* mice were more responsive to stimulation, therefore indicating a relief from immunosuppression [167,168].

A large body of results supports the fact that PlGF binding to VEGFR-1 promotes and stimulates the recruitment and/or activation (e.g., cytokine production) of macrophages in the tumor microenvironment [87,89,169,170,171,172,173,174].

About this, Fischer et al. demonstrated that an anti-PlGF neutralizing antibody inhibits the growth of VEGFR2-inhibitor-resistant tumors by blocking the accumulation of pro-angiogenic macrophages in tumors and by normalizing the count of circulating monocytes, in orthotopic mouse models. The mechanism by which anti-PlGF reduces tumor growth was associated with lesser severe hypoxia and smaller necrotic tumor areas than anti-VEGFR2 treatment [169].

Accordingly, the presence of macrophages within the tumor microenvironment has been associated with enhanced tumor growth and spread, angiogenesis and immunosuppression both in animal models and human cancer [170,171,173,174,175,176,177]. TAMs are also involved in regulating the angiogenic switch [178]. The paradoxical role of TAMs in cancer could be accountable for their capability to polarize as M2-like phenotype, which is characterized by PlGF upregulation.

In this regard, it has been reported that PlGF signaling is able to induce macrophage polarization to a TAM subtype in a model of larynx carcinoma [179]. Such polarization is associated to increase the expression level of MMP9 induced by PlGF. On the other hand, MMP9, which is found to be activated in the PlGF-polarized TAM via transforming growth factor β (TGF-β) receptor signaling activation, in turn upregulated PlGF. In addition, to being involved in the process of metastasis and invasiveness, MMP9 has been reported to mediate immunosuppression of CD8^+^ tumor-infiltrating lymphocytes (TIL) through a proteolytic process of IL-2Rα [180].

Moreover, TAMs with M2-like phenotype express and release Th2 cytokines such as IL-6, IL-10, CCL-22, and TGF-β. This behavior could result in the polarized expression of pro-tumoral functions. Indeed, these cytokines promote the differentiation of monocytes to mature macrophages but block their differentiation to dendritic cells (DC), attenuate the Th1 immune response as well as impair the activity of cytotoxic T lymphocytes and NK cells. [172]. It is also interesting to note that IL-6 and IL-10 have been shown to inhibit maturation of bone marrow progenitors or monocytes into DCs, instead of driving monocytes toward a suppressive phenotype [19].

An additional link between macrophages polarization and PlGF has been attributed to Histidine-rich glycoprotein (HRG). HRG is a heparin-binding plasma protein produced in the liver with anti-inflammatory effects and synthesized by monocytes and macrophages as well. Although the cellular receptor has not yet been identified, HRG is able to bind numerous ligands and to modulate different functions, including immunity and vascularization, by binding to different cells such as endothelial cells, fibroblasts, T cells, and monocyte/macrophage cells [181].

Intriguingly, HRG shows both antiangiogenic and pro-angiogenic activity. The first one is likely mediated through disrupting of endothelial cells cytoskeleton by preventing the ability to migrate and to assemble into vessel structures, even though VEGF-induced cell proliferation is not affected by HRG [182]. On the other hand, the pro-angiogenic activity of HRG is mediated by the binding with high affinity to thrombospondin-1 (TSP-1), which is known to be a potent inhibitory factor of vascular growth, by masking the antiangiogenic epitope of TSP-1 [183]. In addition, HRG is deposited in the tumor stroma, although its levels are decreased in human cancer, both because tumor cells usually express low levels of HRG [184] and because HRG is partially degraded to inactive fragments by tumors [182]. Despite its apparently conflicting effects on angiogenesis, HRG inhibits tumor growth [185] but its precise mechanisms are not yet fully known.

In this regard, Rolny et al. reported that, when HRG is overexpressed (HRG^+^) in a different type of cancer cells, tumors grew slower and metastasized less when implanted in wild-type mice. Moreover, tumor growth was reduced despite persistent TAMs accumulation. By analyzing the cytokines produced by these cells, they found that the exposure of TAMs to HRG downregulated the M2 markers such as IL10, CCL22, and PlGF, while simultaneously elevated M1 markers such as IL6 and CXCL9. Therefore, HRG mediated vascular normalization and increased antitumoral immunity. Indeed, the reduced expression of IL10 and CCL22 could decrease the recruitment of Treg cells and consequently improve DCs and T cells function. Moreover, the increase of CXCL9 could promote CD8^+^ T cells and NK cells infiltration into tumor stroma. Thus, these effects were mediated by re-education of TAMs to relieve the immunosuppressive tumor microenvironment. The mechanism by which HRG influenced TAMs polarization relies largely on downregulation of PlGF. Indeed, the growth of both HRG^+^ and control tumor cells was reduced to the same extent when transplanted in *plgf-/-* mice. These findings indicated that HRG did not further suppress tumor growth in the absence of host-derived PlGF and confirmed that HRG-mediated macrophage polarization is dependent on the suppression of PlGF [171].

In addition, He et al. reported the cross talk between TAM and non-small cell lung cancer (NSCLC) cells via PLGF/VEGFR1. PLGF expressed by NSCLC cells shows a critical role in promoting metastases by triggering macrophage polarization to a TAM subtype, which in turn promotes the growth of NSCLC cells. Moreover, polarized TAM appeared to secrete TGF-β1 to enhance the growth of endothelial cells [186].

A further insight into the understanding the role played by PlGF in the intratumoral immune microenvironment have been added by Incio et al., using a murine pancreatic ductal adenocarcinoma (PDAC) and breast cancer (BC) models orthotopically implanted in obese mice.

They reported that targeting PlGF/VEGFR-1 signaling reprograms the tumor immune microenvironment and inhibits obesity-induced acceleration of tumor progression. Specifically, when PDAC and BC cells were implanted in obese mice showed that tyrosine kinase (TK) deletion of VEGFR1 signaling led to a reduction in tumor progression associated with a shift in tumor cytokine profile and TAMs polarization towards the M1 phenotype. Accordingly, since plasma PlGF, but not VEGF-A, was associated with obesity in PDAC and BC patient samples as well as in mouse models, consistent with this notion, *plgf−/−* null mice reproduced exactly the phenotype of the *vegfr1*-*(TK^-^)* mice on improving the immune environment and reducing tumor growth in the obese setting [174]. These results are further confirmations that TAMs polarization towards the M2 phenotype is mediated by the signaling of PlGF and not VEGF via VEGFR1.

Interestingly, it has been reported by Laoui et al. that TAMs, which are predominant in the hypoxic core of tumor, express MHC-II^low^ while TAMs located outside the hypoxic zones are MHCII^high^. Thus, tumor hypoxia finely adjusts the MHCII^low^ TAMs phenotypes making them less efficient in antigen presentation rather than driving TAMs differentiation [187].

Overall, the effect of PlGF-recruited TAMs in tumor milieu not only promotes tumor growth but also compromises the ability of other immune cells to have inhibitory activities against cancer cells.

#### 6.2.2. Interplay between TAMs and Others Innate Immunity Cells

As mentioned above, the tumor microenvironment is characterized by severe hypoxia associated with high levels of lactate and consequent low pH. As macrophages migrate to accumulate in the most severely hypoxic regions of the tumor tissue, the high amounts of lactate, produced by tumor cells, decrease the activation of pro-inflammatory macrophages; promote the M2 polarization with a consequent reduction of NF-*κ*B activity, which in turn can compromise tumor surveillance by inhibiting of infiltrating T and NK cells [188]. Thus, the reduced ability of the T and NK cells may not only be due to the pro-tumor polarization of the macrophage M2 but essentially to inefficient capability to activate immune alternative circuits.

Noteworthy, NK cells are effector lymphocytes of the innate immune system that control and limit the spread of altered or infected cells and subsequent tissue damage. Consistent with the innate sentinel’s function, NK cells are widespread in lymphoid and non-lymphoid tissues.

Like T cells, NK cells require priming for complete activation, but most of the NK cell priming mechanisms remain unclear. However, during the maturation process, NK cells follow an education step to become both competent to recognize “missing self”, such as poorly immunogenic tumor cells, and “tolerant to self” through MHC class I recognition [189]. Regrettably, most antigens expressed by tumor cells are not exclusively present in cancer cells but rather they are tissue-differentiation antigens also expressed in normal cells.

Despite the exact role of tumor-infiltrating NK cells (TINKs) as well as the correlation between their presence and prognosis in cancer is still unclear, numerous in vitro and in vivo studies have shown that tumor cells are recognized as NK cell targets and that NK cells have a relevant role in the control of tumor development in mice. Indeed, mice depleted of NK cells are more susceptible to methylcholanthrene-induced sarcomas [190], as well as epidemiological studies in humans have shown that low blood NK cell activity is associated with increased cancer risk in adults [191]. On the other hand, although the presence of TINKs may indicate an ongoing immune response against the tumor, many studies have shown that intense NK cells infiltration is associated with advanced disease [192]. In fact, TINKs display a tumor-associated phenotype characterized by decreased cytotoxicity, overexpression of inhibitory receptors and defective expression of activating receptors [191,193,194].

NK cells are also regulatory cells engaged in mutual interactions with other immune cells. Therefore, NK cells may limit or exacerbate immune response; as a result, both cellular interactions and the local environment in which the NK cell resides in may influence its cytotoxic functions. The intensity and the feature of NK cell cytotoxic and cytokines release also depend on the cytokine milieu, as well as on interactions with other cells of the immune system such as macrophages, DCs, and T cells [195,196].

In this regard, Nuñez et al. reported that M2 macrophages were able to limit NK cell effector functions through the decrease of TGF-β secretion and engagement of CD85j. They found a lower percentage of activated NK cells that produced fewer IFN-γ upon co-culture with M2. In addition, when co-cultured with M2 macrophages, CD56^dim^ NK cells displayed lower degranulation and cytotoxic activity than NK cells co-cultured with M1. Additionally, the hyporesponsiveness to degranulation in NK cells was not restored despite the removal of M2. Thus, M2 macrophages restrain NK cell activation and effector functions through different mechanisms, leading to NK cells that display diminished IFN-γ production and impaired degranulation ability [197].

A further relationship between M2 polarized macrophages and NK cells inactivation has been provided by Krneta et al. They reported that in vitro co-cultures of murine NK cells with bone marrow-derived M2-polarized macrophages or TAMs, isolated from spontaneous mouse breast tumors, were able to inhibit the NK cell activation and cytotoxicity against tumor cells. The mechanism of NK cells inhibition required contact between the respective cell types and was due to the production of TGF-β by both M2 and TAMs. In fact, the inhibition of TGF-β was able to restore the cytotoxicity of NK cells in the presence of TAMs. In addition, TAMs induced a CD27^low^CD11b^high^-exhausted NK cell phenotype, consistent with the reduced activation and cytotoxicity observed [198].

In conclusion, these findings unravel an inhibitory circuit. In fact, since PlGF activates the recruitment and polarization of M2 macrophages and, in turn, M2 is able to inhibit the cytotoxic activity of NK cells, it is legitimate to state that PlGF can blunt the cytotoxic activity of NK cells (Figure 1).

#### 6.2.3. Relationship between PlGF and Adaptive Immunity Cells

**Figure 2:**
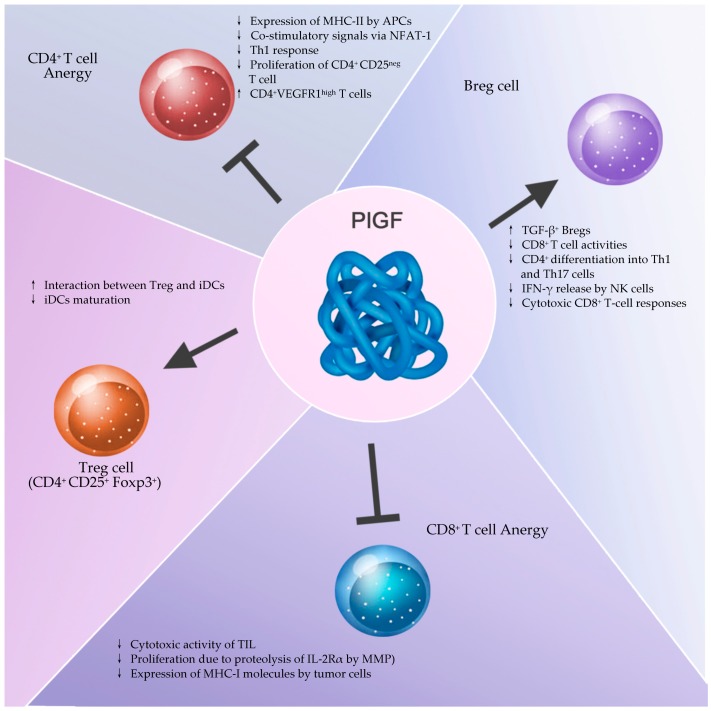
The effects of PlGF on adaptive immune cells. The lack of maturation of dendritic cells in the presence of PlGF prevents the activation of naïve CD4^+^ T cells due to the reduced expression of costimulatory molecules, and results in both the suppression of naïve CD4^+^ T cell proliferation and Th1 polarization. PlGF also stimulates the proliferation of CD4^+^VEGFR1^high^ T cells and TGF-β+ regulatory B cells (Bregs). PlGF, by the induction of tumor-associated macrophages (TAMs) polarization, can mediate immunosuppression of CD8^+^ tumor infiltrating lymphocytes (TIL). Neuropilin-1 (NRP1) receptor on T cells involved in the immunological synapse between Treg cells and immature DCs (iDCs) induces immunosuppression due to delay iDCs maturation. Abbreviations: APCs, antigen presenting cells; MHC, major histocompatibility complex; NFAT-1, nuclear factor of activated T cells-1; IL-2 Rα, IL-2 high affinity receptor.

Although the effects of PlGF on adaptive immune responses include few studies, Lin et al. reported the evidence of immunosuppressive properties of PlGF. They found that PlGF inhibited the activation and maturation of human DCs, differentiated from CD14^+^ monocytes, effectively and rapidly through the NF-*κ*B signaling pathway. PlGF-treated DC resulted in the downregulation of maturation markers CD80, CD86, CD83, CD40, and MHC-II expression as well as the inhibition of IL-12, IL-8, and TNF-α production in response to LPS stimulation, in respect to untreated DC. PlGF inhibited DC maturation through the VEGFR1, and this PlGF-induced DC dysfunction was rescued by anti-human VEGFR1 mAb, while VEGFR2 mAb failed to recover the PlGF-induced inhibition. These results showed that DC inhibition was mediated by PlGF and not by VEGF. In addition, treatment of DC with PlGF resulted in the suppression of *naïve* CD4^+^ T cell proliferation in an allogeneic mixed lymphocyte reaction. The results from this study indicate that PlGF can downregulate type 1 T helper immune responses by modulating the function of DCs [199] (Figure 2).

Interestingly, among the previously identified subsets of CD4^+^ Tregs, it has been reported that CD4^+^VEGFR1^high^ T cells are a novel subset of Tregs that regulate the inflammatory response. Further, CD4^+^VEGFR1^high^ T cells are able to suppress the proliferation of CD4^+^CD25^neg^ T cell as efficiently as CD4^+^CD25^high^ natural Tregs in a contact-independent manner by soluble factor-mediated induction of apoptosis, in a model of lymphopenic mice. These findings strengthen the importance of the role of CD4^+^VEGFR1^high^ T cells in the maintenance of immune homeostasis and are additional evidence for the link between the effects of VEGFR1 mediated signaling and the inadequate immune response in tumors [200] (Figure 2).

Further confirmation of the PlGF effect on the immune response, was reported by Kang et al. using a PlGF-overexpressing transgenic (Tg) mice model. PlGF secretion was upregulated in isolated T-cells, stimulated by activated CD3/CD28, hence suggesting PlGF as a regulator of T-cell differentiation in an autocrine or paracrine manner. In addition, the CD4^+^ T-cells isolated from the spleen of Tg mice indicated greater inflammatory Th1 and Th17 helper T-cell differentiation, thereby emphasizing the role of PlGF in T-cell differentiation and development [201] (Figure 2).

Rising pieces of evidence report, that an increase in Treg lymphocytes infiltrating the tumor, is associated with worse prognosis in ovarian, mammary and gastric tumors [202,203,204] but with a better prognosis in colorectal cancer and in some lymphomas [205,206]. These findings suggest that the function of Tregs in tumors may be context-dependent [207]. However, although the involvement of each Treg subgroup, in addition to classically described CD4^+^CD25^+^Foxp3^+^ Treg cells, Foxp3^neg^ Tr1 cells and Th3 cells, in regulating different aspects of the immune response is still largely elusive, later studies have highlighted that NRP1 allows distinguishing among Treg cell subsets in vivo. Indeed, NRP1 is selectively expressed at high levels only on natural Treg cells (nTreg), which arise in the thymus, but not on inducible Treg cells (iTreg), generated in the periphery through induction of Foxp3 [208].

Furthermore, although the ligand for NRP1 on Th cells is not yet determined, Sarris et al. reported that NRP1 functions as a component of the immunological synapse and promotes prolonged interaction between Treg cells and immature DCs (iDCs). The long contact between *naïve* Th cells with antigen-bearing iDCs results in higher NF-*κ*B transcriptional activity and enhanced cell growth compared to those of control Th cells. These effects prevent an autoimmune response and induce immunosuppression due to delay or, even worse, to the prevention of iDC maturation [209]. 

An emerging body of evidence has recently recognized a role for B cells in modulating the immune response against tumors and lymphoid malignancies. In particular, regulatory B cells (Bregs) are a newly designated subset of B lymphocytes that have been shown to play a pivotal role in regulating immune responses involved in inflammation, autoimmunity and, more recently, cancer [210,211,212,213]. Indeed, Bregs can suppress different cell functions such as T-cell differentiation into Th1 and Th17 cells; pro-inflammatory cytokine production by CD4^+^ effector T cells; IFN-γ release by NK cells via the production of IL-10, TNF-α production by monocytes, and cytotoxic CD8^+^ T-cell responses. Further, through the expression of FasL, Bregs can initiate apoptosis in effector T cells. In addition, Bregs can also promote the differentiation of T cells to Treg through the secretion of anti-inflammatory mediators, such as IL-10 and TGF-β, and thus blunting anti-tumor immune responses [210,211,212,213,214].

In this regard, Han et al., have reported that cells from surgically removed glioma tissue released exosomes carrying PlGF. When purified (CD19^+^ IL-7R^+^ CD45^+^) *naïve* B cells captured the PlGF-containing exosomes from glioma cells, they differentiated into TGF-β^+^ Bregs able to suppress the CD8^+^ T cell activities. Further, the treatment of glioma cells with an anti-PlGF antibody (TB-403), a (PlGF)-specific inhibitor, completely suppressed the expression of the TGF-β, while the exposure of glioma cells to PlGF, upregulated the expression of the TGF-β [215] (Figure 2). This effect mediated by exosomes loaded/carrying with PlGF is a further demonstration of the role played by this growth factor on the immune response against the tumor.

Worthy of note, exosomes are cytoplasmic multivesicular bodies released into the extracellular environment through plasma membrane fusion. The exosomes, being able to stably transfer their content such as proteins, lipids, nucleic acids and miRNA in distant sites, constitute a mode of cross talk between the cells and have been involved in multiple physiological and pathological processes. Apart from tumor cells, different immune cells, including DCs, macrophages, B cells, T cells, and NK cells can also release exosomes [216,217,218,219,220]. For these reasons, tumor-related exosomes are reputed capable of regulating the immune response with a consequent downstream blockade in antitumor immune responses [221] and have been widely implicated in tumor progression [216,220,221,222].

#### 6.2.4. PlGF and Tumor Angiogenesis

Different experimental approaches have shown that PlGF effects on tumor angiogenesis are still enigmatic. Studies based on genetic or pharmacological inhibition showed that PlGF induces vascular endothelial responses in vitro (migration, survival, proliferation) [86,87,114] and affects tumor angiogenesis in vivo [84,172,175]. In addition, the absence of *plgf* has been found to decrease pathological angiogenesis in the adult and to reduce tumor growth in vivo. Furthermore, embryonic stem cells develop small hypervascularized tumors when implanted in *plgf -/-* mice [82,83,86]. PlGF can stimulate vessel growth and maturation directly by affecting endothelial and mural cells, and indirectly by recruiting pro-angiogenic cell types. Nonetheless, the direct role of PlGF in cancer remains unclear. In fact, PlGF overexpression has been shown to result in tumor growth promotion or inhibition, perhaps due to context-dependent effects of this growth factor in tumor progression [169,223,224,225,226,227,228]. In addition, studies in transgenic mice revealed that the angiogenic activity of PlGF is restricted to pathological conditions since PlGF expression is low to undetectable in most tissue in normal health [84,85]. However, despite several studies have indicated that PlGF is a pro-angiogenic factor, others have reported that PlGF upregulation, by promoting the formation of less active VEGF/PlGF heterodimers, decreases the angiogenic activity of cancer cells [229,230]. However, some of these studies have been performed in vivo using the PlGF-1 isoform, which, in addition to being absent in the mouse, has a less powerful biological effect than PlGF-2 [223,229,230]. In addition, one study has been performed employing xenograft tumor cells that did not express VEGFR1 [223]. Conversely, in vitro studies have shown that the responsiveness to PlGF of cancer cells necessarily relies on the expression of VEGFR1 [231].

A possible explanation of the direct PlGF effects in pathological angiogenesis derives from a recent study by Clegg and Mac Gabhann. Their computational analysis on in vivo VEGFR activation by multiple co-expressed ligands have predicted that, as opposite to the “ligand-shifting hypothesis”, VEGF and PlGF do not compete for receptor binding at physiological concentrations, though PlGF is predicted to slightly increase VEGFR2 phosphorylation when over-expressed by 10-fold [232]. This result further supports that PlGF exerts its effects when it is strongly upregulated as occurs during inflammation and cancer.

However, PlGF signaling can indirectly contribute to the tumor angiogenic switch by upregulating the expressions of other angiogenic factors (VEGF-A, FGF2 and MMPs) [85,172,226]. PlGF promotes the recruitment and maturation of angiogenesis-competent myeloid progenitors, of circulating endothelial precursor cells to growing sprouts and collateral vessels and of VEGFR1^+^ hematopoietic progenitor cells and macrophages to growing tumor sites. These effects thus contribute to vasculogenesis, neoangiogenesis and lymphangiogenesis [71,91,93,94,96,110,114,115,116,169].

#### 6.2.5. PlGF and Cancer Progression

Accumulating evidence has suggested that PlGF might be a useful prognostic marker for cancer progression. Plasma PlGF levels are upregulated and correlate with tumor grade and survival in different type of tumors. PlGF levels are also elevated in BCR-ABL1^+^ chronic myeloid leukemia (CML) and PlGF produced by bone marrow stromal cells (BMSCs) by close contact with leukemia cells aggravates disease severity. Indeed, CML cells take advantage of their own growth by inducing BMSCs to upregulate PlGF, which in turn promotes CML proliferation, in part independently of BCR-ABL1^+^ signaling. In this setting, anti-PlGF treatment prolonged survival of imatinib-sensitive and -resistant CML mice and added to the anti-CML activity of imatinib [88].

Kaplan et al. have described that bone marrow-derived cells (most macrophage-lineage cells), during tumor progression, migrate and infiltrate the tissues establishing cellular clusters before the arrival of tumor cells in a VEGFR1-dependent manner, and these clusters provide a “pre-metastatic niche” for the original tumor. In fact, the inhibition of VEGFR1 function or the removal of VEGFR1^+^ cells from the bone marrow abrogates the formation of these pre-metastatic clusters and prevents tumor metastasis. The evidence that, in human tissues from patients with malignancy, there were increased cellular clusters of VEGFR1^+^ cells in common sites of metastasis, before tumor spread, has confirmed these results. Moreover, VEGFR1^+^ cellular clusters express VLA-4 and the hematopoietic progenitor marker c-Kit. Thus, the mechanism by which VEGFR1 favors this process is mediated by VLA-4 binding to fibronectin, which in turn, alters the local microenvironment with the release of soluble Kit-ligand by upregulating the expression of MMP9, to support the newly introduced cells that express c-Kit [233]. These and other more recent pieces of evidence imply that PlGF/VEGFR1 signaling, as well as being involved in recruiting and migration of macrophage-lineage cells, is also involved in cancer metastasis [90,173,234,235]. Additionally, PlGF/VEGFR1 signaling has been shown to inhibit apoptosis, to induce survival and chemoresistance through Akt and NF-*κ*B in tumor cells and to enhance cells motility through ERK 1/2 signaling [236,237,238,239,240]. Nonetheless, although the presence of VEGFR-1 is necessary for a number of tumors to respond to the anti-PlGF therapy [231], the direct inhibition of VEGFR-1 does not always replicate the effects obtained with the anti-PlGF therapy in other tumor models [241], thus suggesting an alternative signaling mechanism for PlGF, independently from the presence of VEGFR-1. In this regard, it has been shown that the co-receptors NRPs can replace, in some circumstances, the lack of VEGFR1 signaling when binds PlGF.

Interestingly, it has been reported that the high-level expression of NRP1 in tumor cells and the upregulation of PlGF in the cerebellar stroma are required for the growth and spread of medulloblastoma in mice. This phenomenon correlates with poor overall survival in patients. Further, this study reports that PlGF promotes tumor cell survival through NRP1 and not VEGFR1. The blockade of PlGF/NRP1 signaling results in direct antitumor effects and increases mouse survival by demonstrating that NRP1 is involved in transduction of downstream PlGF signaling, independently from the presence of VEGFR1 [172,235].

## 7. Anti-PlGF Targeting Strategies

Recent studies have highlighted some of the mechanisms by which PlGF can promote the metastatic process [242,243,244]. In this regard, it has been reported that PlGF expression is positively correlated with the low expression level of E-cadherin and the high expression level of vimentin in cervical cancer and that these levels correlate with the metastatic ability of the tumor. Indeed, PlGF promotes molecular changes of epithelial-mesenchymal transition (EMT) through ERK/MAPK signaling pathway in cervical cancer cells [245]. PlGF also promotes metastases of ovarian cancer through suppression of microRNA-543 (miR-543), which in turn, promote the translation of MMP7 [246]. Moreover, Li et al. reported that PlGF/c-MYC/miR-19a axis promotes metastasis and stemness in gallbladder cancer (GBC). They found that PlGF expression levels were higher in GBC tissues than in normal adjacent tissues and were associated in poor prognosis in GBC patients. The in vitro assay showed that exogenous PlGF enhanced the migration, invasion, and tumorsphere formation of GBC cells. Conversely, knockdown of PlGF decreased the aggressive phenotype of GBC cells. Mechanistically, exogenous PlGF upregulated miR-19a expression through the activation of c-MYC with a positive pairwise correlation among PlGF, c-MYC, and miR-19a expression in GBC tissues [247]. Again, PlGF overexpression in oral squamous carcinoma cells (OSCC) is parallel with the increased expression of MMP9, while anti-PlGF treatment of OSCC decreases the expression of MMP9. Conversely, the reduction of MMP9 does not affect the levels of PlGF [248].

Owing these properties, PlGF has been proposed as a drug target for anti-angiogenic therapy [169,249,250] as PlGF plasma levels and intratumoral expression have been found to correlate with tumor stage, recurrence, metastasis, and survival in different types of cancer. [88,239,243,244,245,246,247,248,250,251] (Table 1). Indeed, in a mouse model, an anti-PlGF antibody was able to inhibit tumor growth without affecting healthy vessels by reducing the infiltration of angiogenic macrophages and by decreasing severe tumor hypoxia, thus preventing the switch-on of the angiogenic rescue program responsible for resistance to VEGF-receptors inhibitors therapy [169] (Table 1).

In this regard, TB-403 (RO5323441), a humanized recombinant IgG1 monoclonal antibody, directed to the receptor-binding site of PlGF, has demonstrated that targeting PlGF can result in significant inhibition of tumor growth and metastasis in preclinical studies. However, up to now, only a few clinical trials concerning TB-403 are available. TB-403 has been shown to significantly inhibit tumor growth in xenograft tumor models and it is thought to act in a pleiotropic manner and with a complementary mechanism to VEGF(R) inhibitors [251,254]. The proposed mechanisms of action of TB-403 are the reducing of intratumoral macrophage recruitment accompanied by a normalizing effect on the increased levels of circulating monocyte in experimental tumor models of pancreatic and colon cancer [252] and the induction of TGF-β^+^ Bregs differentiation able to suppress the CD8^+^ T cell activities [215].

In the first-in-human study, healthy male subjects were given single doses of TB-403 or placebo. No serious adverse events (SAEs) or adverse events (AEs) related to treatment were reported and no differences in safety profile were observed among the different dose levels of TB-403 [253,255].

A phase I dose-escalation study with TB-403 was performed by Lassen et al. in patients with advanced solid tumors. They enrolled twenty-three patients with a different type of tumors. Stable disease was observed in six of 23 patients at different dose levels. Mild (grade 1 or 2) were the most frequently reported adverse events (AEs). There were no serious AEs in the study, and none of the AEs led to withdrawal compared to what happens instead with the anti-VEGF therapy [256]. However, the sample size was too small to evaluate the effectiveness of the treatment although no dose relationship was observed [253,256] (Table 1).

Oude Munnink et al., in two human tumor xenografts model, used labeled TB-403 in order to trace the tumor uptake and localization. They found that PlGF-expressing hepatocellular cancer (HCC) and renal cell carcinoma (RCC) without detectable human PlGF expression xenografts showed a time- and dose-dependent accumulation of labeled TB-403. In HCC xenograft-bearing mice, TB-403 exhibited mainly intratumoral distribution, while in RCC xenograft-bearing mice TB-403 accumulated mainly in the tumor microenvironment. In addition, pretreatment of HCC xenograft-bearing mice reduced TB-403 tumor uptake to nonspecific level although TB-403 was significantly accumulated in the tumor microenvironment [252] (Table 1). These pieces of evidence suggest that TB-403 could also be valuable in those tumors whose cells do not express a detectable level of PlGF, due to its ability to interfere with the tumor microenvironment.

A recent study, which analyzed the pharmacokinetic interaction (PK) between different anticancer drugs, reported that TB-403, when given in combination with bevacizumab, shows unexpected PK characteristics. In fact, the serum concentrations of TB-403 were 50% higher than the results observed when it was individually administered. This decreased clearance may indicate the presence of drug-drug interaction between TB-403 and bevacizumab, which involves a target-trapping mechanism via VEGFR1 [250] (Table 1).

Other studies on the effects of TB-403 on macrophage recruitment and activation and on antitumor immune response may provide therapeutic benefit in certain stages of tumor progression and should be further examined in future studies.

## 8. Conclusive Remarks

Although many aspects of the adaptive immunity, in which the PlGF may be involved have not yet been fully explored, PlGF has revealed a direct role in the innate immune response by favoring M2 macrophages recruitment and polarization, MDSC recruitment and effects on DC and NK cells, it is reasonable to admit an impact on the adaptive immune response. In fact, PlGF amplifying the effect of VEGF may induce a state of immunosuppression (a condition that occurs in tumor-bearing hosts) always associated with high serum levels of VEGF and PlGF. There are several pieces evidence showing that PlGF has an impact on adaptive immunity: i) PlGF prevents the maturation of dendritic cells (similarly to VEGF), which represent the first step of the adaptive response. ii) Acting on NK, PlGF would interrupt the crosstalk between NK and dendritic cells just necessary for triggering the adaptive response. iii) During pregnancy, PlGF increases Treg cells and the cytokines associated with the polarization of this T cell subtype. In pregnancy, the mother’s immune system has to tolerate the persistence of paternal alloantigens without affecting anti-infectious immune responsiveness. Thus, elevated levels of PlGF would be important in inducing maternal-fetal tolerance. iv) The coreceptor NRP1 is constitutively expressed on the surface of CD4^+^CD25^+^ Treg cells and can even transmit alone, in some circumstances, signaling when binds PlGF. v) Bregs glioma-derived exosomes contain PlGF that suppresses the glioma-specific CD8^+^ T cell activities.

Overall, these shreds of evidence could suggest future research directions and contribute to most effective anti-cancer strategies.

## Figures and Tables

**Table 1:** Preclinical and clinical studies resulting in PlGF downregulation or upregulation.

Preclinical Trials					
Model	Tumor Type	PlGF Targeting	Angiogenesis	Tumor Progression	Reference
C57BL/6 Plgf−/−	ES-cell–derived tumors	PlGF ko	↓	↓	[86]
C57BL/6 mice	T241 fibrosarcoma	PlGF-1 overexpression	↓	↓	[223]
SCID mice.	A549 lung, HCT116 colon, and U87-MG glioblastoma	PlGF upregulation	↓	↓	[229]
Flt1TK−/−, Plgf−/−, ob/ob, C57BL/6 mice	PAN02 pancreatic tumors, E0771breast cancer	Inhibition of PlGF signaling	↓	↓	[174]
Female C57BL/6, BALB/c, Swiss mice. mice PlGF-/-	B16.F10, CT26, EL-4 and Panc02 xenograft	PlGF ko and Anti-PlGF mAb 5D11D4	↓	↓	[169]
Vegfr1 tk−/− mice	B16F/10; LLC; EL4 tumor cell lines xenograft	Anti-PlGF mAbs C9.V2 and 7A10	↔	↔	[223]
C57BL/6 mice	B16F/10; LLC; EL4 tumor cell lines xenograft	Anti-PlGF mAbs C9.V2 and 7A10	↔	↔	[224]
*nu/nu* mice	MDA-MB-435 human pancreatic DanG HCC xenograft	Anti-PlGF mAb 16D3, Anti-PlGF mAb 5D11D4	↓	↓	[226]
Balb/c, C57Bl/6 WT/WT and PlGF−/−	Bcr-Abl1^+^ xenograft	Anti-PlGF mAb 5D11D4	↓	↓	[88]
*Smo/Smo* medulloblastoma and *flt1*^TK−/−^ mice	D283-MED and D341-MED medulloblastoma xenograft	Anti-PlGF mAb TB-403 (RO5323441) anti-PlGF mAb 5D11D4	↓	↓	[172]
Athymic nude mice (BALB-c/Ola HSD-fox)	Hepatocellular carcinoma (HCC) and renal cell carcinoma (RCC) xenograft	TB-403 (RO5323441)	↓	↓	[252]
**Clinical trials**					
Phase I dose-escalation study	23 patients with different advanced cancer	TB-403 (RO5323441)	↓ 6/23	↓ 6/23	[253]
Phase 1 dose-escalation study	22 patients with recurrent glioblastoma	TB-403 (RO5323441) + bevacizumab	It does not appear to improve effectiveness obtained with single-agent bevacizumab	progression-free 3.5 months, overall survival 8.5 months	[251]
